# Giant cystic malignant pheochromocytoma invading right hepatic lobe: report on two cases

**DOI:** 10.1590/S1516-31802008000400008

**Published:** 2008-07-03

**Authors:** Sergio Renato Pais Costa, Nivaldo Marques Cabral, Ademir Torres Abhrão, Ricardo Borges da Costa, Lilian Mary da Silva, Renato Arioni Lupinacci

**Keywords:** Pheochromocytoma, Neuroendocrine tumors, Hepatectomy, Adrenalectomy, Case reports [publication type], Feocromocitoma, Tumores neuroendócrinos, Hepatectomia, Adrenalectomia, Relatos de casos [tipo de publicação]

## Abstract

**CONTEXT:**

Cystic pheochromocytomas are uncommon neuroendocrine tumors that originate from the adrenal medulla. Differing from the more frequent solid pheochromocytomas, which produce catecholamines and present adrenergic syndrome, cystic pheochromocytomas may not produce these. Their symptoms are generally associated with an abdominal mass or even pain, particularly if the mass attains large dimensions. Similarly, radiological diagnosis may also be difficult. Right-side lesions may be confounded with cystic hepatic tumors or even retroperitoneal sarcomas with cystic areas, using radiological methods. Sometimes, there may be a preoperative diagnosis of malignancy. Invasion of organs in this region (i.e. liver or kidney), or even the presence of a large retroperitoneal mass (of uncertain origin) with which multiple organs are involved, may be indicative of malignant origin.

**CASE REPORT:**

Two cases of giant cystic pheochromocytoma that invaded the right hepatic lobe are described. These presented as abdominal masses. Both cases were malignant. They were treated by radical right nephrectomy plus right hepatectomy.

## RESUMO

**CONTEXTO:**

Feocromocitomas císticos são tumores neuroendócrinos raros oriundos da glândula adrenal. Ao contrário dos feocromocitomas sólidos (mais comuns), que usualmente produzem catecolaminas e se manifestam com uma síndrome adrenérgica, os feocromocitomas císticos podem não produzi-las. Por conseguinte, seus sintomas habituais estão relacionados à presença de massa abdominal ou dor, mormente quando atingem grandes dimensões. Devido a essa singularidade, o seu diagnóstico, inclusive radiológico, pode ser complexo. Lesões à direita podem ser confundidas com tumores hepáticos císticos ou mesmo sarcomas de retroperitônio com áreas císticas pelos exames de imagem. Invasão de órgãos contíguos (fígado, rim) ou mesmo massa retroperitoneal de grandes dimensões envolvendo múltiplos órgãos (sem uma origem identificada) podem ser indicativos de sua natureza maligna.

**RELATO DE CASOS:**

Os autores relatam dois casos de feocromocitoma cístico gigante com invasão do lobo hepático direito. Esses tumores apresentavam quadro clínico de massa abdominal. Ambos os casos eram de feocromocitomas malignos e foram tratados com nefrectomia radical direita e hepatectomia direita em monobloco.

## INTRODUCTION

Pheochromocytomas are rare catecholamine-secreting tumors that originate from chromaffin cells in the adrenal medulla. They are solid and well-vascularized neoplasms, as seen by radiological imaging.^1-3^ Very rarely, they may present as predominantly cystic masses. To date, only a few cases of purely cystic pheochromocytomas have been reported in the worldwide literature.^4^ Two cases of malignant cystic pheochromocytomas are presented.

### Case 1

A healthy 46-year-old Caucasian man was referred for evaluation of an abdominal mass. Physical examination revealed a slightly tender 30-cm mass in the upper right quadrant. A computed tomography (CT) scan revealed a 30-cm predominantly cystic mass. This mass seemed to originate from the right hepatic lobe and apparently invaded the right kidney (the right adrenal gland was not identified). The laboratory tests were all within normal limits (carcinoembryonic antigen, CA19-9, CA125 and alpha-fetoprotein levels and 24-hour assays for catecholamines and their metabolites).

**Figure 1:**
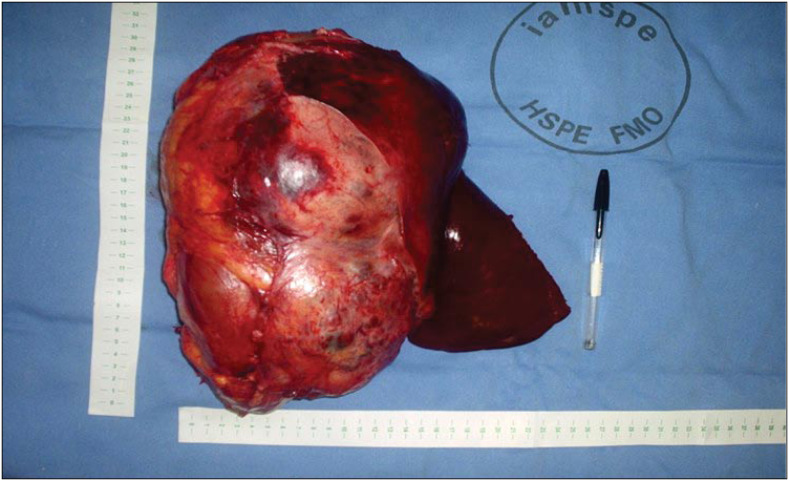
(Case 1) – Surgical specimen from *en-bloc* radical right nephrectomy plus right hepatectomy.

Surgical exploration was indicated. The surgical access was a right thoracoabdominal approach.^5^ On exploration, a 30-cm fixed round well-vascularized retroperitoneal cystic mass that involved the right hepatic lobe, right kidney and right adrenal was found. Intraoperative analysis was unable to elucidate the tumor origin. *En bloc* right radical nephrectomy with right hepatectomy was performed (Figure 1).

The patient's postoperative course was uncomplicated until the sixth postoperative day. Although a complete preoperative evaluation of cardiac risks had been carried out, the patient suddenly suffered extensive myocardial infarction and died. Postoperative 24-hour assays for catecholamines and their metabolites presented normal levels. Histological analysis showed a solid-cystic adrenal neuroendocrine tumor that invaded both the liver and the right kidney (both surgical margins were disease-free). The result from immunohistochemical staining confirmed the presence of malignant pheochromocytoma (with high expression of telomerase).

### Case 2

**Figure 2:**
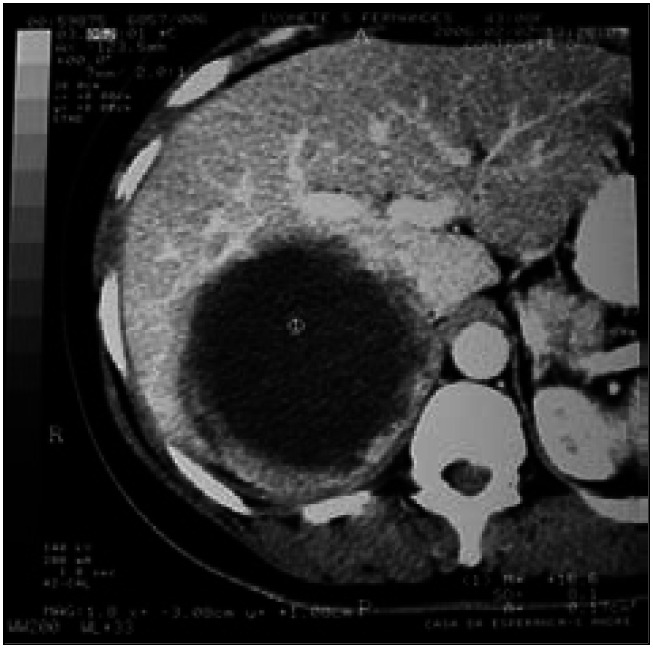
(Case 2) – Predominantly cystic retroperitoneal tumor that involved right hepatic lobe (computed tomography scan).

A healthy 43-year-old Caucasian woman was referred for evaluation of an abdominal mass (positive medical history of both papillary thyroid carcinoma and ductal breast carcinoma). Physical examination showed a slightly tender 18-cm mass in the upper right quadrant. A CT scan revealed a 16-cm predominantly cystic mass with a thick irregular wall. This mass was retroperitoneal (with an adrenal site) and appeared to be invading both the right hepatic lobe and right kidney (Figure 2). The radiological diagnosis was compatible with malignant adrenal tumor. The laboratory tests were all within normal limits (24-hour assay for catecholamines and their metabolites).

Surgical exploration was indicated. The surgical access was a bilateral subcostal incision with medial extension (Mercedes-Benz incision). During exploration, a fixed round well-vascularized cystic mass that involved the right hepatic lobe, common bile duct, right kidney and right adrenal was found. The tumor originated from the right adrenal gland. *En bloc* right radical nephrectomy with right hepatectomy and partial common bile duct resection was performed.

**Figure 3:**
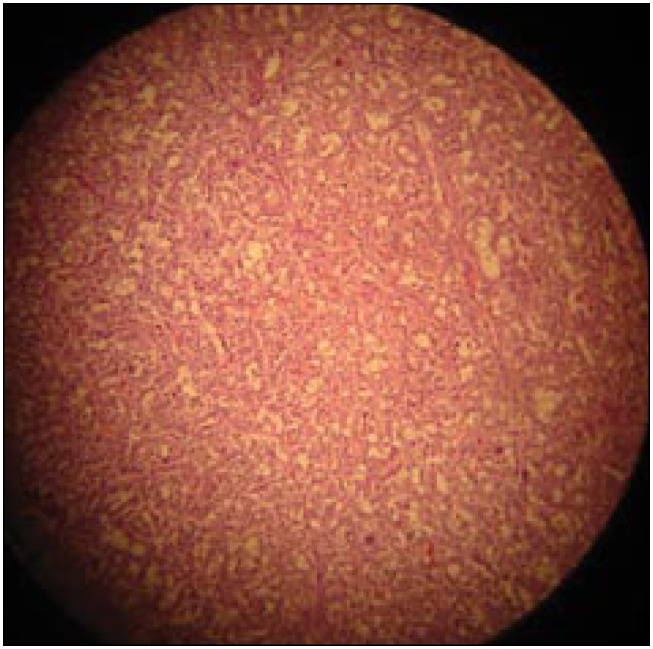
(Case 2) – Pheochromocytoma (hematoxylin-eosin, 10 × 10): *en-bloc* polygonal cell neoplasm.

**Figure 4:**
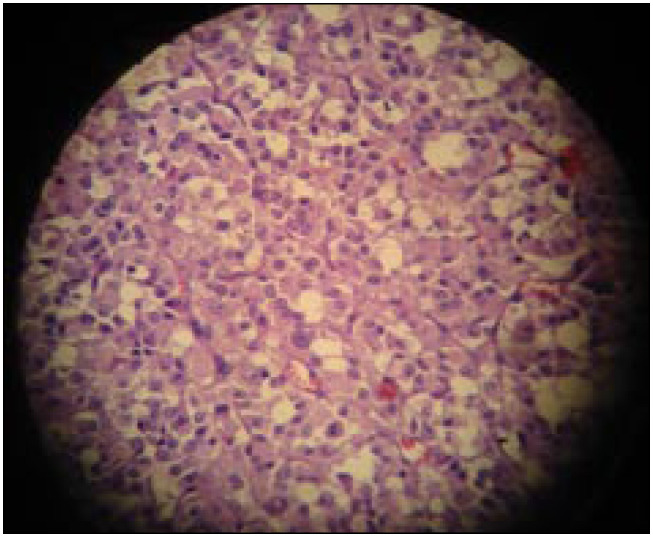
(Case 2) – Malignant pheochromocytoma (hematoxylin-eosin, 10 × 40): intense cellular pleomorphism and atypical nuclei.

The patient's postoperative course was uncomplicated, and she was discharged home on the sixteenth postoperative day. The histological analysis showed a cystic adrenal neuroendocrine tumor that invaded both the liver and the right kidney (surgical margins were disease-free). The histological findings were compatible with pheochromocytoma (Figures 3 and 4). The results from immunohistochemical staining confirmed the presence of malignant pheochromocytoma (with high expression of telomerase).

After a postoperative asymptomatic period of one year, she began to present hypertension plus headache. The 24-hour assays for catecholamines and their metabolites presented normal levels. She probably developed primary systemic arterial hypertension during this follow-up period. Monitoring using nuclear magnetic resonance (NMR) imaging showed three hepatic nodules (hepatic segments II and IV). She then underwent a metaiodobenzylguanidine (MIBG) scan, which was positive. Consequently, she was administered iodine-131 MIBG therapy. She presented excellent clinical and radiological responses to this.

## DISCUSSION

Pheochromocytomas are highly vascular tumors, and most are unilateral and solitary.^6,7^ They are rarely malignant. As with other neuroendocrine tumors, the diagnosis of malignancy is not primarily based on cytological characteristics, but is defined by the presence of local invasion or metastatic disease.^8,9^ The pathological distinction between benign and malignant pheochromocytomas is unclear. Malignant tumors tend to be larger and weigh more,^9^ as we observed in the present case, although this is not an absolute criterion. The only absolute criterion for malignancy is the presence of secondary tumors at sites where chromaffin cells are not usually present, and the presence of visceral metastases. Additionally, detection of high expression of telomerase and hTERT and high proliferative activity, as measured by means of immunohistochemistry using the MIB-1 antibody, provides strong support for the hypothesis that the pheochromocytoma case is biologically malignant.^9^

Concomitantly, as we observed in both of our cases, the presence of radiological findings like invasion of contiguous organs or even hepatic metastasis may suggest malignancy.^8,9^ The classical symptoms of pheochromocytomas are hypertension associated with palpitations, headache and diaphoresis.^8^ Once a pheochromocytoma is suspected, initial screening for 24-hour urine metanephrine and vanillylmandelic acid levels is appropriate, and thus the tumor is located using radiological methods (CT or NMR).^2,3^ Since most tumors that produce catecholamines avidly incorporate iodine-131 MIBG, MIBG scintigraphy may be helpful for locating small pheochromocytomas.^8,9^ Once a diagnosis of functioning pheochromocytoma has been established, the preoperative preparation includes alpha-adrenergic blockade (phenoxybezamine). If tachycardia develops, beta-adrenergic blocking agents (propranolol) are added.^9^

On the other hand, for purely cystic pheochromocytomas, the classical symptoms may not be found and there may even not be any elevation of the urinary levels of catecholamine metabolites.^4,10^ Therefore, the use of preoperative alpha or beta-blockade is not usually necessary. This absence of classical signs and symptoms may complicate the preoperative diagnosing of pheochromocytomas.^4,10^ There may even be confusion with giant hepatic cystic neoplasms such as described by Wu et al.^11^

There are 18 case reports of purely cystic pheochromocytomas in the worldwide literature.^4,12^ Among these, seven patients did not present hypertension and the cystic pheochromocytoma was not diagnosed preoperatively.^4,11-16^ The clinical findings may be abdominal masses or even pain, as observed in previously reported cases.^4,11^

In summary, multivisceral resection of locally advanced cystic malignant pheochromocytomas (with invasion of contiguous organs) should be considered for low-risk individuals. Additionally, both pre and postoperative care are advisable. Cardiac risks must particularly be controlled for. There has been some interest in treating metastatic lesions with therapeutic doses of iodine-131 MIBG, which may present good results (case 2). Long-term survival may be attained: the five-year survival rate for malignant pheochromocytomas is around 43%.^17^
